# Progressive cerebellar degeneration revealing Primary Sjögren Syndrome: a case report

**DOI:** 10.1186/s40673-016-0056-0

**Published:** 2016-10-19

**Authors:** Emna Farhat, Mourad Zouari, Ines Ben Abdelaziz, Cyrine Drissi, Rahma Beyrouti, Mohamed Ben Hammouda, Fayçal Hentati

**Affiliations:** 1 Department of Neurology, National Institute Mongi Ben Hamida of Neurology, Rue Jébal Lakhdhar La Rabta Bab Saâdoun 1007, Tunis, Tunisia; 2 Department of Radiology, National Institute Mongi Ben Hamida of Neurology, Tunis, Tunisia

**Keywords:** Sjӧgren syndrome, Ataxia, Cerebellar atrophy, Degenerative, Antineuronal antibodies, Cyclophosphamide

## Abstract

**Background:**

Cerebellar ataxia represents a rare and severe complication of Sjӧgren syndrome (SS), especially with a progressive onset and cerebellar atrophy on imaging.

**Case presentation:**

We report the case of a 30-year-old woman, with a past history of dry eyes and mouth, who presented a severe cerebellar ataxia worsening over 4 years associated with tremor of the limbs and the head. Brain MRI showed bilateral hyperintensities on T2 and FLAIR sequences, affecting periventricular white matter, with marked cerebellar atrophy. Complementary investigations confirmed the diagnosis of primary SS (pSS). The patient was treated by methylprednisolone, Cyclophosphamid and Azathioprine. Her clinical and radiological states are stabilized after 2 years of following. Primary cerebellar degeneration is extremely rarely associated with pSS. Few cases of isolated cerebellar ataxia or belonging to a multifocal disease were reported in the literature, most of them characterized by an acute or rapidly progressive onset. Cerebellar atrophy was described in only three patients. There have been few clarifications of the pathogenesis of the neurological manifestations in pSS. Treatment is based on corticosteroids and immunosuppressive agents with no consensus of a specific therapy.

**Conclusions:**

Cerebellar ataxia due to pSS may exceptionally mimic a degenerative cerebellar ataxia, especially when the onset is progressive, which represents the particularity of our observation. The role of brain MRI and antibodies remains important for the differential diagnosis.

## Background

Sjögren syndrome (SS) is a chronic autoimmune disease involving mainly salivary and lacrimal glands, characterized by mononuclear cell infiltration of the exocrine glands, which leads to typical xerophtalmia and xerostomia. Neurological manifestations occur in approximately 20 % of patients [1–4]. Since peripheral nervous system’s (PNS) involvement has been widely described, several studies reported variable prevalence of central nervous system (CNS) manifestations ranging from 2.5 and 60 % of all primary SS (pSS) patients [1, 5, 6]. Although ataxia due to pSS has also been described [7–10], marked cerebellar atrophy associated with pSS has rarely been reported. We report the case of a 30-year-old woman who presented a progressive cerebellar syndrome with marked cerebellar atrophy on imaging revealing a pSS.

## Case report

A 40-year-old Tunisian female, without any past history of dysimmune disorder or arthralgia, developed progressive unsteadiness of gait worsening over 4 years. She had no familial history of gait disturbance. She complains also about tremor of limbs and head appearing since 1 year. On initial neurological examination, she was unable to walk, with a severe cerebellar syndrome including dysarthria and ataxia of four limbs and trunk. Motor power and sensory examinations were normal. Her deep-tendon reflexes were normoactive, and her plantar responses were flexor bilaterally. Bilateral rest and intention tremor of the four limbs and head was also noticed. She had no nystagmus, no bladder dysfunction, no parkinsonism and no cognitive impairment. General examination was otherwise normal. During her hospitalization, she complained about dry eyes and mouth.

Complete blood count, serum electrolytes, creatinine, glucose, coagulation tests, liver functional tests, lacticodeshydrogenase, blood sedimentation rate, lipids, and creatine kinase were normal.

**Fig. 1:**
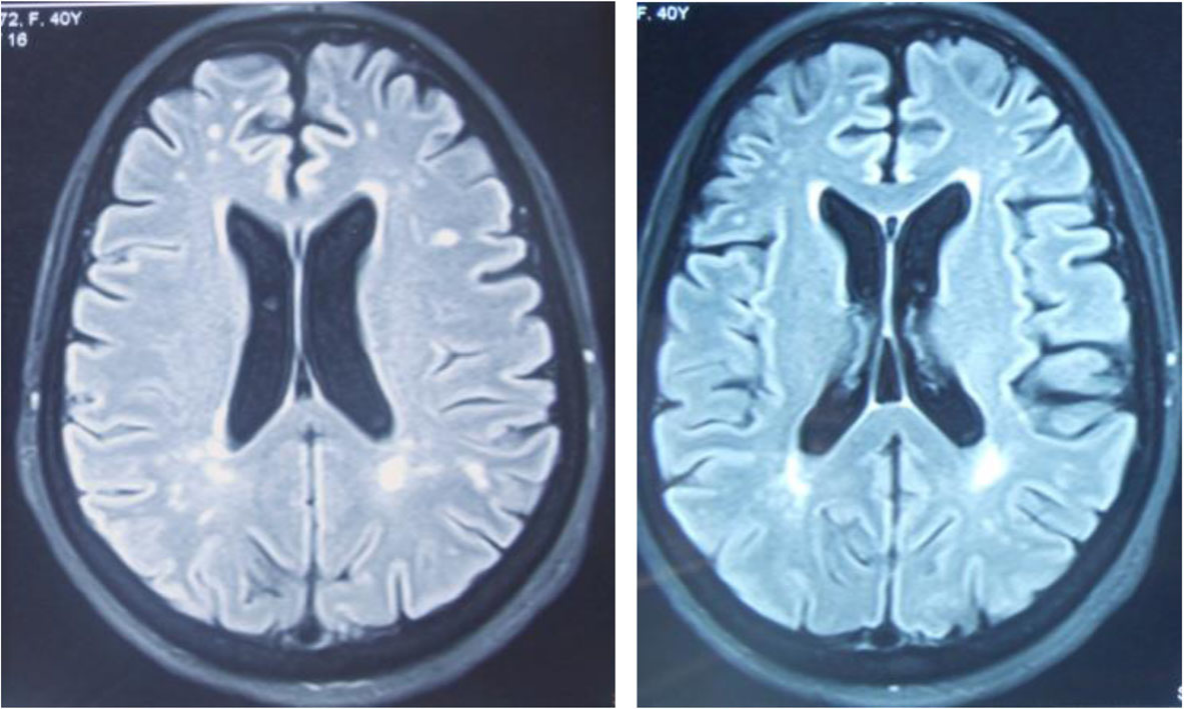
Brain MRI FLAIR sequences showing bilateral hyperintensities, affecting periventricular regions predominantly in the subcortical white matter

**Fig. 2:**
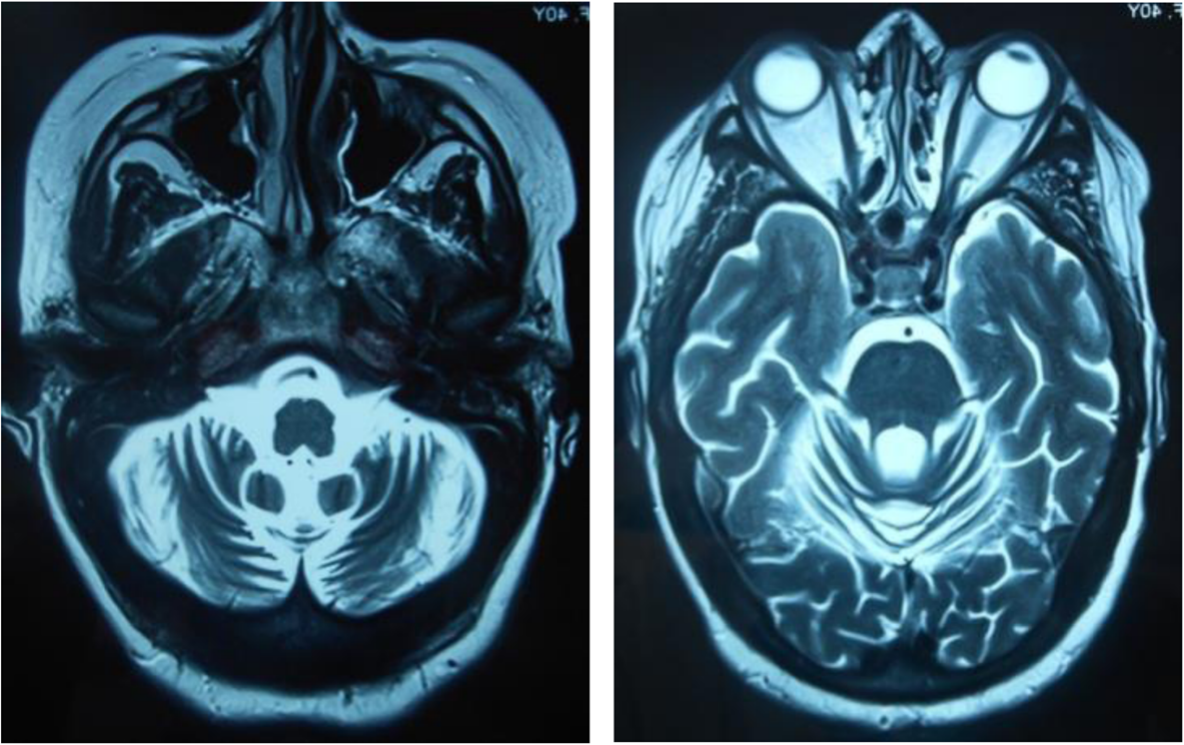
Brain MRI T2 sequences showing marked cerebellar atrophy with an enlarged fourth ventricle and cisterna magna, without signal abnormities of the cerebellum

Brain magnetic resonance imaging (MRI) showed bilateral hyperintensities on T2 and FLAIR sequences, affecting periventricular white matter, predominantly in the subcortical white matter. No gadolinium enhancement was found (Fig. 1). Marked cerebellar atrophy without signal abnormalities was notified (Fig. 2). Spinal MRI was normal. Visual evoked potential and electromyography were both normal. Toxic causes of cerebellar ataxia were ruled out by the interrogatory.

The cerebrospinal fluid contained 01 cell/ mm3 and 70 mg/dl of protein, and the glucose level was not decreased. There was no oligoclonal bands. Laboratory tests were positive for anti-Ro-SSA antibodies. Anti-nuclear antibodies were slightly positive (1/100) and speckled on immunofluorescence, DNA antibodies were negative. Assays for ss-B (anti-La), anti-phospholipids (cardiolipin and β2 glycoprotein), anti-nuclear ribonucleoprotein (RNP), anti-Smith (Sm), anti-histone, anti PM-Scl, anti-PCNA, anti-Scl70, anti-centromere, anti-Jo1, anti-ribosomal and anti-nucleosome antibodies were all negative. Rheumatoid factor was also negative. HIV and hepatitis tests were negative. Paraneoplasic antibodies (anti-Hu, anti-Ri, anti-Yo, anti-PNMa2 and anti-CRMP5), anti-GAD, anti-gliadin, anti-transglutaminase, anti-thyroglobulin and anti-thyroid peroxidase antibodies were not detected. Anti-VGCC antibodies were not tested. The break up time test confirms the xerophtalmia. Salivary glands biopsy was not performed because of the important head tremor. The diagnosis of pSS with neurological complications was retained according to the 2002 American European classification criteria [11].

Genetic tests for Friedreich ataxia and ataxia with vitamin E deficiency were negative.

The patient was treated by intravenous methylprednisolone (1 g/day), administered for 5 days followed by maintenance therapy with oral methylprednisolone (1 mg/kg/day) continued for 3 months, and then progressively decreased. She received Cyclophosphamid once per month for 6 months. Normocardil (60 mg/day) and Meprobamate (600 mg/day) were also administered for tremor. There was no significant clinical improvement, but her state did not worsened after 2 years of follow up. A second brain MRI, made 3 months after she received sixth dose of Cyclophosphamid, showed the same stable lesions.

## Discussion

In contrast to the uniform features of the PNS complications of pSS, CNS abnormalities are associated with a much wider spectrum of manifestations. In the largest cohort of 431 pSS patients published in 2010, the most frequent picture of CNS involvement was a recurrent subacute encephalopathy characterized by memory loss, cognitive dysfunction, visual disturbances and dizziness [7]. The first description of cerebellar syndrome in association with pSS was by Attwood and Poser in 1961 [12]. Since then, few cases of isolated cerebellar ataxia or belonging to a multifocal disease were reported in the literature, most of them characterized by an acute or rapidly progressive onset [7–10, 13].

Primary cerebellar degeneration is extremely rarely associated with pSS. There are few reports of cerebellar degeneration associated with different autoimmune diseases, especially with systemic lupus erythematous [14, 15], and Neuro-Behçet’s disease [16, 17]. To our knowledge, only three cases of pSS causing cerebellar atrophy have been reported [9, 18, 19], two of them having positive neuronal antibodies [9, 18]. The first case of cerebellar ataxia with cerebellar atrophy and negative neuronal antibodies was reported by Kim et al. in 2012 [19]. The patient was a 46-year-old woman who presented a rapidly progressive onset ataxia 3 months before her first examination. She had isolated cerebellar syndrome, marked cerebellar atrophy with an enlarged fourth ventricle and cisterna magna on brain MRI, but without any signal abnormalities on cerebral or cerebellar parenchyma. Brain PET revealed decreased glucose metabolism in the bilateral cerebellum. However, our patient exhibited progressive onset (4 years before examination), associated rest and intention tremor, and multiple white matter hyperintensities in subcortical and periventricular areas on imaging. In the two cases, there was no significant improvement by treatment.

Movement disorders have been rarely described in association with pSS, and are less classic that in systemic lupus erythematous. There are few reports of chorea, dystonia, athetosis and tremor [20]. Extrapyramidal syndrome has been more frequently reported [21–24].

There have been few clarifications of the pathogenesis of the neurological manifestations in pSS. In PNS involvement, vasculitis and perivascular cell invasion are the most common findings [25, 26]. Some reports have also described the same mechanism in CNS pathology of SS, with subsiding angitis and necrotising vasculitis of small vessels [27–29]. Interestingly, other reports suggested a nonvasculitic pathological mechanism of the CNS damage in SS [30–33]. The latest autopsy case published by Yaguchi et al. in 2008 was about a 40-year-old woman diagnosed with an acute encephalomyelopathy due to a pSS. Neuropathological examination revealed multifocal lesions in the cervical spinal cord and medulla, along with scattered perivascular lymphocytic infiltration. In addition, there was demyelination, spongy change and axonal swelling in the white matter, but no remarkable vasculitic changes were seen in the CNS [34].

This observation confirms the possibility that the main pathological mechanism of CNS damage in SS is not necessarily related to vasculitis. Axonal degeneration, necrotic lesions and perivascular lymphocytic cuffing may explain the non-response to corticosteroid treatment with a fatal evolution in some cases.

The course of the disease of neuro-Sjogren can be MS-like with relapsing-remitting modality [1, 7], or progressive such the case of our patient. In Massara’s paper, it was proved that CNS involvement may even precede clinical diagnosis of SS by many years, with patients misdiagnosed as multiple sclerosis (MS) fulfilling the diagnosis criteria [7].

To date, there is no consensus of a specific therapy the management of Sjögren’s syndrome with CNS involvement. Many kinds of treatments have been used including steroids, immunoglobulins, plasmapheresis, and D-penicillamine [21, 25, 35–37]. Our patient was treated by high doses of methylprednisolone associated with Cyclophosphamid without improvement but also no worseness in her neurological abnormalities.

## Conclusion

Cerebellar ataxia represents a rare and severe complication of pSS. It also represents a diagnostic challenge for the clinician especially when preceding the classic glandular symptoms of the disease, fact that may delay the recognition of SS and heavily affect the outcome. The differential diagnosis between pSS with CNS involvement and classical MS may be sometimes very difficult. The clinician must follow the course of the disease, repeating the look for clinical manifestations and laboratory tests which orient to this particular diagnosis. Cerebellar ataxia due to pSS may exceptionally mimic a degenerative cerebellar ataxia, especially when the onset is progressive, which represents the particularity of our observation. The role of brain MRI and antibodies remains important for the differential diagnosis.

## Abbreviations

CNS: Central nervous system
MRI: Magnetic resonance imaging
MS: Multiple sclerosis
PNS: Peripheral nervous system
pSS: primary Sjӧgren syndrome
SS: Sjӧgren syndrome

## References

[1] Delalande S, Seze J, Fauchais AL, Hachulla E, Stojkovic T, Ferriby D (2004). Neurologic manifestations in primary Sjogren syndrome: a study of 82 patients. Medicine (Baltimore).

[2] Rogers SJ, Williams CS, Roman GC (2004). Myelopathy in Sjogren’s syndrome: role of nonsteroidal immunosuppressants. Drugs.

[3] Atherine L (2000). Neurological manifestations in Sjogren’s syndrome. Arch Neurol.

[4] Alexander EL, Aminoff MJ, Goetz CG (1998). Neurologic disease in Sjogren’s syndrome. Handbook of Clinical Neurology.

[5] Mauch E, Völk C, Kratzsch G, Krapf H, Kornhuber HH, Laufen H (1994). Neurological and neuropsychiatric dysfunction in primary Sjögren’s syndrome. Acta Neurol Scand.

[6] Alexander E (1992). Central nervous system disease in Sjögren’s syndrome. New insights into immunopathogenesis. Rheum Dis Clin North Am.

[7] Massara A, Onazza S, Castellino G, Caniatti L, Trotta F (2010). Central Nervous system involvement in Sjo¨gren’ sindrome: unusual, but non unremarkable-clinical, serological characteristics and outcomes in a large cohort of Italian patients. Rheumatology.

[8] Wong S, Pollock AN, Burnham JM, Sherry DD, Dlugos DJ (2004). Acute cerebellar ataxia due to Sjögren syndrome. Neurology.

[9] Owada K, Uchihara T, Ishida K, Mizusawa H, Watabiki S, Tsuchiya K (2002). Motor weakness and cerebellar ataxia in Sjögren syndrome--identification of antineuronal antibody: a case report. J Neurol Sci.

[10] Collison K, Rees J (2007). Asymmetric cerebellar ataxia and limbic encephalitis as a presenting feature of primary Sjögren’s syndrome Journal of Neurology. November.

[11] Vitali C, Bombardieri S, Jonsson R, Moutsopoulos HM, Alexander EL, Carsons SE, Weisman MH (2002). Classification criteria for Sjögren’s syndrome: a revised version of the European criteria proposed by the American European Consensus Group. Ann Rheum Dis.

[12] Attwood W, Poser CM (1961). Neurologic complications of Sjögren’s syndrome. Neurology.

[13] Chen Y-W, Lee K-C, Chang I-W, Chang C-S, Hsu S-P, Kuo H-C (2013). Sjogren’s syndrome with acute cerebellar ataxia and massive lymphadenopathy: a case report. Acta Neurol Taiwan.

[14] Manto MU, Rondeaux P, Jacquy J, Hildebrand JG (1996). Subacute pancerebellar syndrome associated with systemic lupus erythematosus. Clin Neurol Neurosurg.

[15] Shimomura T, Kuno N, Takenaka T, Maeda M, Takahashi K (1993). Purkinje cell antibody in lupus ataxia. Lancet.

[16] Gardner RC, Schmahmann JD (2008). Ataxia and cerebellar atrophy--a novel manifestation of neuro-Behçet disease?. Mov Disord.

[17] Hirose M, Ikeuchi T, Hayashi S, Terajima K, Endo K, Hayashi T (2006). A possible variant of neuro-Behçet disease presenting chronic progressive ataxia without mucocutaneo-ocular symptoms. Rheumatol Int.

[18] Terao Y, Sakai K, Kato S, Tanabe H, Ishida K, Tsukamoto T (1994). Antineuronal antibody in Sjögren’s syndrome masquerading as paraneoplastic cerebellar degeneration. Lancet.

[19] Kim MJ, Lee MC, Lee J-H, Chung J (2012). Cerebellar degeneration associated with Sjögren’s syndrome. J Clin Neurol.

[20] Govoni M, Padovan M, Rizzo N, Trotta F (2001). CNS involvement in primary Sjögren’s syndrome: prevalence, clinical aspects, diagnosis assessment and therapeutic approach. CNS Drugs.

[21] Nishimura H, Tachibana H, Makiura N, Okuda B, Sugita M (1994). Corticosteroidresponsive parkinsonism associated with primary Sjogren’s syndrome. ClinNeurol Neurosurg.

[22] Visser LH, Koudstaal PJ, Van de Merwe JP (1993). Hemiparkinsonism in a patient with primary Sjogren’s syndrome. A case report and a review of the literature. Clin Neurol Neurosurg.

[23] Creange A, Sedel F, Brugieres P, Voisin MC, Degos JD (1997). Primary Sjögren’s syndrome presenting as progressive parkinsonian syndrome. Mov Disord.

[24] Walker RH, Spiera H, Brin MF, Olanow CW (1999). Parkinsonism associated with Sjogren’s syndrome: three cases and a review of the literature. Mov Disord.

[25] Mori K, Iijima M, Koike H (2005). The wide spectrum of clinical manifestations in Sjogren’s syndrome-associated neuropathy. Brain.

[26] Dyck PJ, Dyck PJB, Engelstand J, Dyck PJ, Thomas PK (1993). Pathologic alterations of nerves. Peripheral Neuropathy.

[27] Kaltreider HB, Talal N (1969). The neuropathy of Sjogren’s syndrome: trigeminal nerve involvement. Ann Intern Med.

[28] Alexander GE, Provost TT, Stevens MB, Alexander EL (1981). Sjorgen’s syndrome: central nervous system manifestations. Neurology.

[29] Rutan G, Martinez AJ, Fieshko JT, Van Thiel DH (1986). Primary biliary cirrhosis, Sjogren’s syndrome, and transverse myelitis. Gastroenterology.

[30] Ichikawa H, Ishihara K, Fujimoto R (2004). An autopsied case of Sjogren’s syndrome with massive necrotic and demyelinating lesions of the cerebellar white matter. J Neurol Sci.

[31] Caselli RJ, Boeve BF, Scheithauer BW, O’Duffy JD, Hunder GG (1999). Nonvasculitic autoimmune inflammatory meningoencephalitis (NAIM): a reversible form of encephalopathy. Neurology.

[32] Caselli RJ, Scheithauer BW, Bowles CA, Trenerry MR, Meyer FB, Smigielski JS (1991). The treatable dementia of Sjorgen’s syndrome. Ann Neurol.

[33] Josephs KA, Rubino FA, Dickson DW (2004). Nonvasculitic autoimmune inflammatory meningoencephalitis. Neuropathology.

[34] Yaguchi H, Houzen H, Kikuchi K, Hata D, Ura S, Takeda T, Yabe I, Sasaki H (2008). An autopsy case of Sjögren’s syndrome with acute encephalomyelopathy. Inter Med.

[35] Chai J, Logigian EL (2010). Neurological manifestations of primary Sjögren’s syndrome. Curr Opin Neurol.

[36] Kassan SS, Moutsopoulos HM (2004). Clinical manifestations and early diagnosis of Sjogren syndrome. Arch Intern Med.

[37] Ramos Casals M, Tzioufas AG, Stone JH, Siso A, Bosch X (2010). Treatment of primary Sjogren syndrome: a systematic review. JAMA.

